# Clinical outcomes of pipeline embolization devices with shield technology for treating intracranial aneurysms

**DOI:** 10.3389/fneur.2022.971664

**Published:** 2022-11-14

**Authors:** Chao Luo, Lide Jin, Jigen Dong, Zaixiang Fu, Erheng Liu, Shi Yin, Lipeng Jian, Pengren Luo, Bo Liu, Wei Huang, Shuai Zhou

**Affiliations:** ^1^Department of Neurosurgery, The Affiliated Hospital of Kunming University of Science and Technology, Kunming, China; ^2^Department of Neurosurgery, School of Medicine, Second Affiliated Hospital, Zhejiang University, Hangzhou, China; ^3^College of Clinical Medicine, Jilin University, Changchun, China; ^4^Medical Faculty, Kunming University of Science and Technology, Kunming, China

**Keywords:** flow diverters, pipeline embolization device with shield technology, pipeline shield, intracranial aneurysm, endovascular therapy

## Abstract

**Introduction:**

As a common endovascular treatment for intracranial aneurysms, the pipeline embolization device (PED) is considered a standard treatment option, especially for large, giant, wide-necked, or dissecting aneurysms. A layer of phosphorylcholine biocompatible polymer added to the surface of the PED can substantially improve this technology. This PED with shield technology (pipeline shield) is relatively novel; its early technical success and safety have been reported. We conducted a systematic literature review with the aim of evaluating the efficacy and safety of the pipeline shield.

**Methods:**

We searched the PubMed, Embase, and Cochrane databases, following the preferred reporting items for Systematic Reviews and Meta-Analysis (PRISMA) guidelines.

**Results:**

We selected five prospective and two retrospective studies for review. A total of 572 aneurysms were included; of these, 506 (88.5%) were unruptured. The antiplatelet regimens were heterogeneous. The rate of perioperative and postoperative complications was 11.1% [95% confidence interval (CI): 6.5–18.9%]. The adequate occlusion rate at 6 months was 73.9% (95% CI: 69.1–78.7%). The adequate occlusion rate of more than 12 months was 80.9% (95% CI: 75.1–86.1%). The mortality rate was 0.7% (95% CI: 0.2–1.5%). Subgroup analyses showed that aneurysm rupture status had no effect on aneurysm occlusion rate, patient morbidity, or mortality.

**Conclusion:**

This review demonstrates the safety and efficacy of the pipeline shield for treating intracranial aneurysms. However, direct comparisons of the pipeline shield with other flow diverters are needed to better understand the relative safety and effectiveness of different devices.

## Introduction

Flow diverters (FDs) enable the application of endovascular therapy for intracranial aneurysms in an increased number of indications. The utilization of FDs has become the preferred treatment option for various types of aneurysms (1–3). Despite their relatively recent development, numerous FDs have been introduced for clinical use. Currently available coating FDs include the pipeline embolization device (PED) with shield technology (referred to as the pipeline shield), derivo embolization device (DED), and p64/p48 MW HPC (Table 1). The pipeline shield incorporates a phosphorylcholine surface coating (4), which is a third-generation PED. It has been shown to reduce intimal hyperplasia (5) and increase early neointimal growth in preclinical studies (6). In *ex vivo* (4) and *in vitro* studies (7, 8), the pipeline shield significantly reduced thrombogenicity in comparison with other FDs. As a new therapeutic technique for intracranial aneurysms, the efficacy of complications associated with the pipeline shield remains unclear, and there is currently no relevant literature that summarizes existing findings. Therefore, this meta-analysis aimed to explore the efficacy and safety of the pipeline shield in treating intracranial aneurysms.

**Table 1:** Comparison of pipeline shield and other surface-coated FDs.

	**Pipeline shield**	**p64/p48 MW HPC**	**DED**
Basic information	Medtronic, 2014	Phenox, 2017	Acandis, 2016
Description (implant section of each device)	A self-expanding mesh cylinder braided from Cobalt-Chromium alloy wires.	A tubular vascular implant that consists of 48 interwoven nitinol wires which are filled with a platinum core.	24 Nitinol wires with radiopaque platinum core looped at the end, with a 48-wire braid.
Coating description	3 nm thick covalently bound phosphorylcholine surface modification.	Glycan-based multilayer hydrophilic polymer coating.	50 nm thin oxide and oxynitride layer.
The mechanism of surface coating	Phosphorylcholine is a major component of the outer membrane of erythrocytes, thus reducing platelet adhesion and activation.	Inhibits initial platelet adhesion mediated by GPIIb/IIIa binding to surface-adsorbed fibrinogen.	Reduces friction during delivery and expansion, thus reducing thrombogenicity.

DED, Derivo Embolization Device; FDs, Flow Diverters; PHC, Hydrophilic Polymer Coating.

## Methods

### Search strategy

We searched the PubMed, Embase, and Cochrane databases to identify studies using the pipeline shield for treating intracranial aneurysms. We used the following search terms: “flow diverter,” “pipeline embolization device,” “PED,” “shield technology,” “surface modification,” and “aneurysm.” We followed the applicable Preferred Reporting Items for Systematic Reviews and Meta-Analyses (PRISMA) guidelines (9). We reviewed literature published between device inception and March 2022 and carefully screened the search results to select studies that were particularly relevant to pipeline shield devices in the neurointerventional field.

### Selection criteria

**Figure 1:**
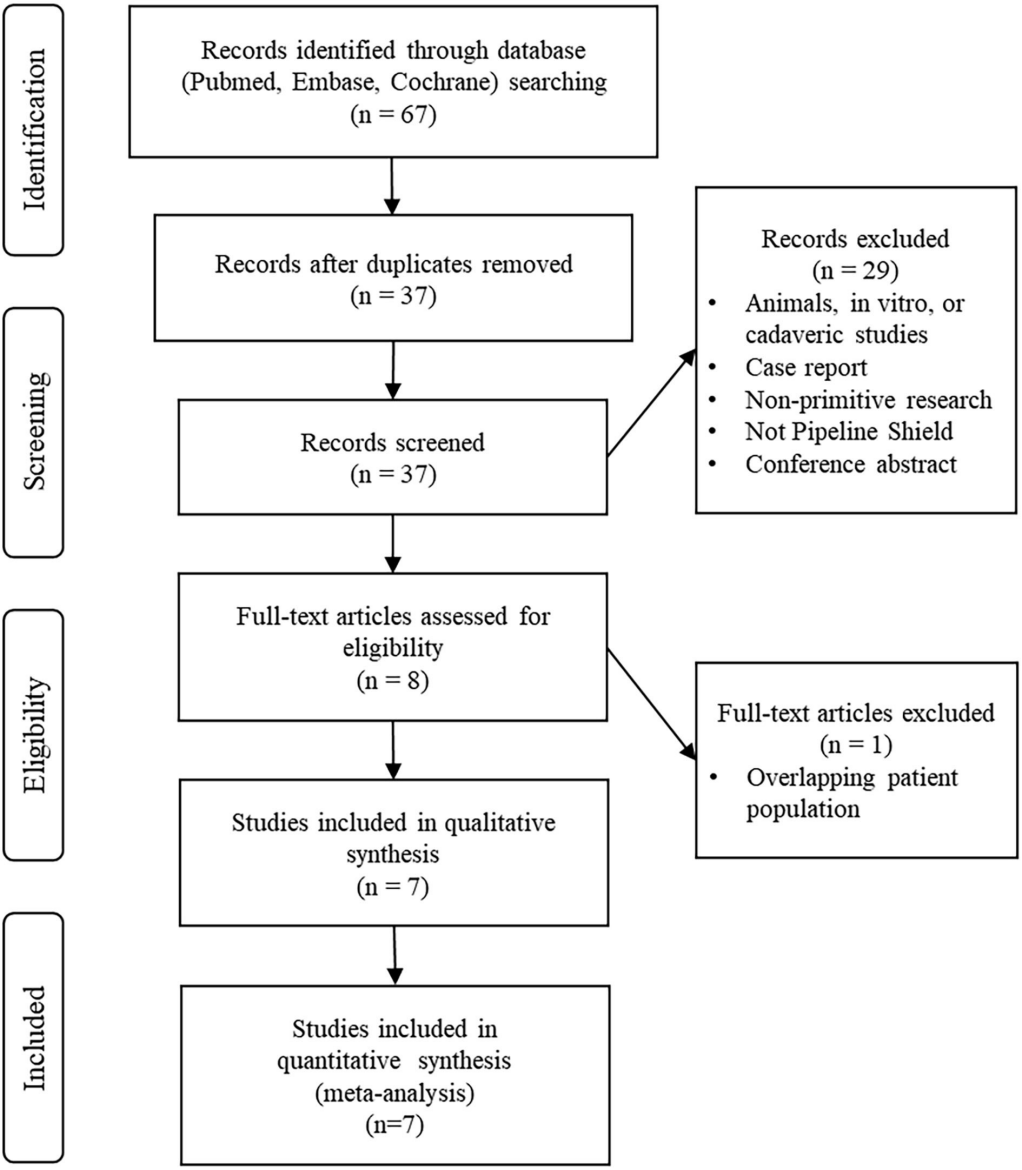
Preferred Reporting Items for Systematic Reviews and Meta-Analyses (PRISMA) flowchart.

For this review, we included all English-language articles on the use of the pipeline shield for treating intracranial aneurysms. Case reports were excluded. Animal, *in vitro*, and cadaveric studies were excluded. We also excluded non-primitive research and conference abstracts. We assessed the center and time frame of included studies with the aim of excluding articles with overlapping cohorts and identifying the most recent and complete studies. We included studies on pipeline shield devices for treating intracranial aneurysms and pooled data on aneurysm occlusion rates, procedural complications, and mortality. The initial search results and screening process are shown in a PRISMA-based (9) flowchart (Figure 1).

### Data selection

We extracted the following data from the included studies: the number of patients, sex ratio, mean age, total number of aneurysms, proportion of ruptured aneurysms at presentation, sizes and neck width of aneurysms, shapes of aneurysms (i.e., blister, fusiform, pseudoaneurysm, or dissecting), locations of aneurysms, devices per aneurysm, mortality rates, morbidity rates, adequate occlusion rate, antiplatelet regimens, and usage of detachable devices.

### Statistical analysis

**Figure 2:**
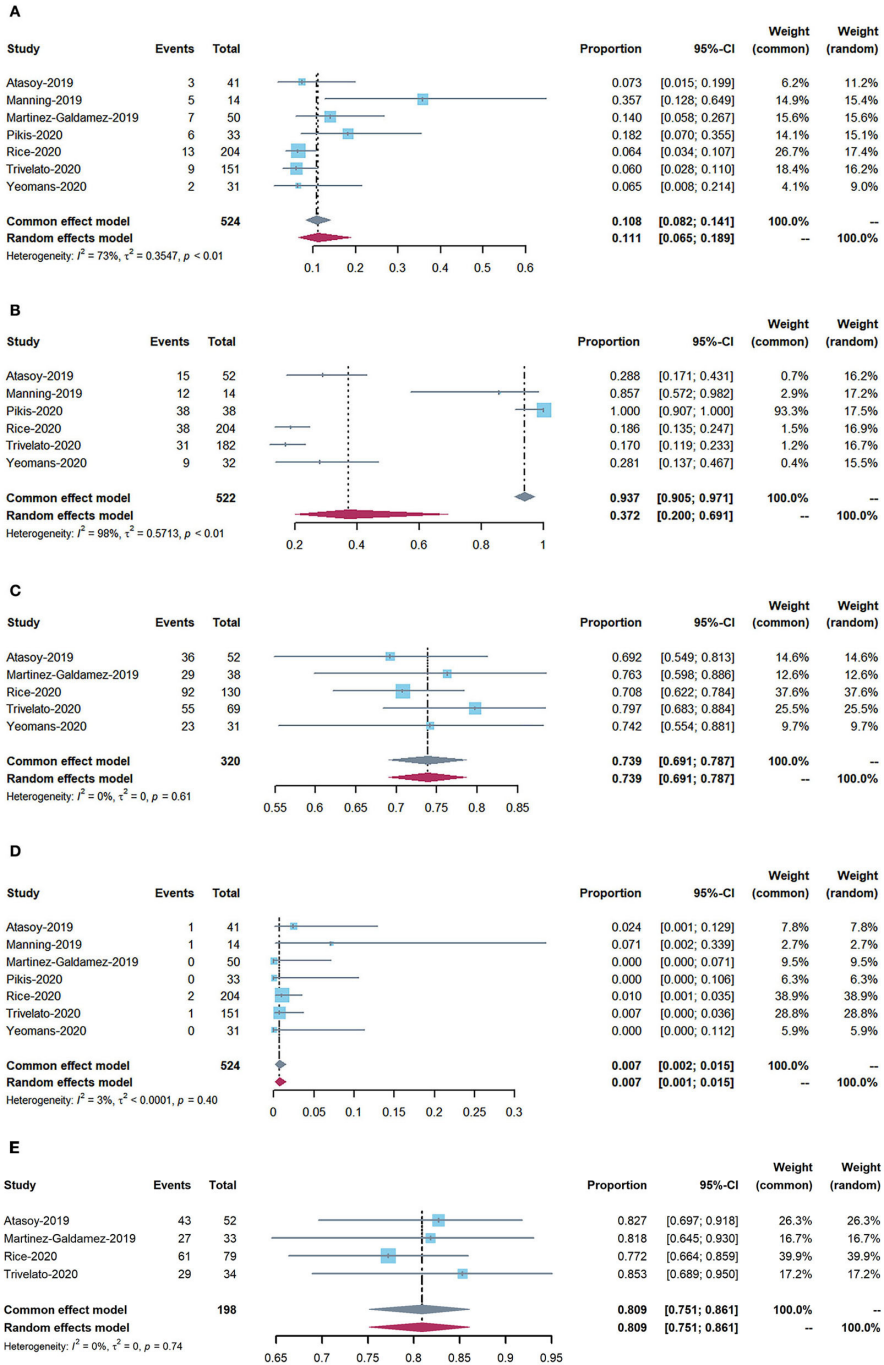
Forest plots: **(A)** periprocedural and postoperative complications; **(B)** use of adjunctive coiling; **(C)** adequate occlusion at 6-month follow-up (defined as Raymond–Roy class 1, O'Kelly–Marotta grade D, or Kamran grade 4); **(D)** mortality; and **(E)** adequate occlusion rate of more than 12 months follow-up.

We used the R package “META” (https://cran.r-project.org) to analyze the acquired data. We calculated proportions across studies and performed meta-analyses using fixed- and random-effects (RE) models for the weighted estimation of the overall rates of each outcome of interest (i.e., periprocedural and postoperative complications, adequate occlusion, and mortality). We also estimated 95% confidence intervals (CIs) and event rates for each outcome. I^2^ statistics were used to assess statistical heterogeneity between studies. For data with I^2^ heterogeneity values >50%, RE models were used. Forest plots were generated based on the proportions and estimated overall rates (Figure 2). Subgroup analyses were conducted using Stata 14.0.

## Results

The preliminary search results contained 67 articles, 30 of which were duplicates. Ultimately, seven articles were selected for further analysis.

### Study characteristics

The characteristics of all included studies are presented in Table 2. Of the seven studies, two were retrospective (10, 17) and five were prospective (12–16). Adjunctive coiling was used in six studies, two of which also used adjunctive balloons. One study used the pipeline shield exclusively. A total of 524 patients with 572 intracranial aneurysms were included. A total of 11.5% of the aneurysms had ruptured before treatment. Most aneurysms were in the anterior rather than posterior circulation (92.1 vs. 7.9%). Aneurysm morphology was identified for all 572 aneurysms: 87.9% were saccular, with the remainder being fusiform, dissecting, blister, or pseudoaneurysms. Table 2 details aneurysm body diameter, neck dilation extent, and parent artery data.

**Table 2:** Characteristics of each study included in our review.

**Study, year**	**No. of patients**	**Age (mean years), sex (% F)**	**Design**	**No. of aneurysms, status**	**Aneurysm sizes**	**Neck width**	**Fusiform, dissecting, pseudoaneur-ysm or blister (%)**	**Circulation (%)**	**Locations (%)**	**No. of the usage of the pipline shield device**				**Adjunctive** **devices (%)**		**Mortality rate (%)**	Morbidity rate^*^(%)	**Adequate occlusion rate (%)**
										**One device**	**Multiple devices**	**Unsuccessful**	**Devices per aneurysm**	**Coiling**	**Balloon**			
Atasoy et al. (10)	41	56, 68.3%	Retrospective study	52 unruptured	60.8% <10 mm 34.6% 10–25 mm 3.8% ≥ 25 mm	5.0, 1.0–21.0 mm (Mean, Range)	5.80%	Anterior circulation:88.5% Posterior circulation:11.5%	3.8% ICA C4 55.7% ICA paraophthalmic segment 23.1% ICA C7 3.8% ICA terminal segment 1.9% MCA M1 5.8% BA 3.8% VA 1.9% PCA	1 device per aneurysm: 41	2 devices per aneurysm;2	One device	0.86	28.8%(15/52)	-	2.4% (1/41)	7.3% (3/41)	69.2% (36/52) at 6 months; 82.7% (43/52) at 18 months.
Manning et al. (11)	14	63, 85.7%	Retrospective study	14 ruptured	35.7%>10 mm 64.3% ≤ 10 mm	Unknown	50%	Anterior circulation:57.1% Posterior circulation:42.9%	21.4% MCA M1 14.3% ACA A1/A2 7.1% AChA 7.1% AcommA 7.1% ACA A2 21.4% VA 14.3% PICA 7.1% PcommA	Unknown	Unknown	-	1.2	85.7% (12/14)	-	7.1% (1/14)	35.7% (5/14)	50.0% (7/14) patients with immediate aneurysm occlusion
Martinez-Galdamez et al. (12)	50	53, 82%	Prospective study	50 unruptured	76% < 10 mm 22% 10–25 mm 2% ≥ 25 mm	Unknown	2.00%	Anterior circulation:94% Posterior circulation:6%	94% ICA 6% VA	Unknown	Unknown	Three devices	1.12	-	-	0	14% (7/50)	76.3%(29/38) at 6 months; 81.8%(27/33) at 12 months.
Pikis et al. (13)	33	54.4, 81.8%	Prospective study	31 unruptured 7 ruptured	68.4% < 10 mm 21.1% 10–25 mm 3% ≥ 25 mm	Unknown	7.90%	Anterior circulation:92.1% Posterior circulation:7.9%	92.1% ICA 7.9% BA	1 device per aneurysm: 35; 1 device for three aneurysms: 1	2 devices per aneurysm: 1	-	0.97	100%(38/38)	-	0	18.18% (6/33)	Not pursued.
Rice et al. (14)	204	54.8, 81.4%	Prospective study	166 unruptured 38 ruptured	50% < 7 mm 33.8% 7–13 mm 13.7% 13–25 mm 2.5% ≥ 25 mm	4.6 ± 2.39 mm(mean ± SD)	4.90%	Anterior circulation:93.6% Posterior circulation:6.4%	1.5% ACA A1 2.5% ACA A2 5.9% AcommA 1.0% MCA M1 0.5% MCA M2 6.4% MCA bifurcation 1.0% ICA C1 0.5% ICA C2 1.5% ICA C3 3.9% ICA C4 8.8% ICA C5 41.2% ICA C6 19.1% ICA C7 6.4% VA V4	1 device per aneurysm: 177	2 devices per aneurysm;23	Four devices	1.1	18.6% (38/204)	10.8% (22/204)	1.0% (2/204)	6.4% (13/204)	70.8% (92/130) at 6 months; 77.2% (61/79) at 12 months.
Trivelato et al. (15)	151	52.7, 79.5%	Prospective study	175 unruptured 7 ruptured	The mean aneurysm size was 7.0 mm; 27 (14.8%) aneurysms were large, and 7 (3.8%) were giant.	4.1 ± 2.1 mm(mean ± SD)	7.10%	Anterior circulation:93.4% Posterior circulation:6.6%	6.5% ACA 11.0% Cavernous 8.2% Communicating 10.4% MCA 53.8% Paraophthalmic 11.1% Other	1 device per aneurysm: 177	2 devices per aneurysm: 4; 3 devices per aneurysm: 1	-	1.03	17%(31/182)	11.5% (18/182)	0.66% (1/151)	6.0% (9/151)	79.7% (55/69) at 6 months; 85.3% (29/34) at 12 months.
Yeomans et al. (16)	31	58.8, 84.1%	Prospective study	32 unruptured	50% < 10 mm 41% 10–25 mm 9% ≥ 25 mm	5.9 ± 3.0 mm(mean ± SD)	100%	Anterior circulation:94% Posterior circulation:6%	3.1% ACA 15.6% ICA C4 6.3% HA 3.1% MCA bifurcation 18.8% ICA paraophthalmic 46.9% PcommA 3.1% Distal BA 3.1% Proximal BA	1 device per aneurysm: 29	2 devices per aneurysm: 3	-	1.09	28.1% (9/32)	-	0	6.5% (2/31)	74.2% (23/31) at 6 months.

ACA, anterior cerebral artery; AcommA, anterior communicating artery; AChA, anterior choroidal artery; A1/A2, first/second segment; BA, basilar artery; C1, cervical segment; C2, petrous segment; C3, lacerum segment; C4, cavernous segment; C5, clinoid segment; C6, ophthalmic segment; C7, communicating segment; F, female; HA, hypophyseal artery; ICA, internal carotid artery; MCA, middle cerebral artery; M1, pre-bifurcation segment; M2, post-bifurcation segment; No, number; PCA, posterior cerebral artery; PcommA, posterior communicating artery; PICA, posterior inferior cerebellar artery; SD, standard deviation; VA, vertebral artery; V4, intradural segment;

* Perioperative and postoperative 1 year such as ischemic/hemorrhagic stroke and other complications.

### Complications and mortality

The rate of perioperative and postoperative complications was 11.1% (95% CI: 6.5–18.9%). The overall mortality rate was 0.7% (95% CI: 0.2–1.5%).

### Angiographic outcomes

The rate of adequate occlusion at 6-month follow-up was 73.9% (95% CI: 69.1–78.7%). The adequate occlusion rate of more than 12 months was 80.9% (95% CI: 75.1–86.1%). Moreover, the rate of adjunctive coiling use was 37.2% (95% CI: 20–69.1%).

### Subgroup analysis

**Figure 3:**
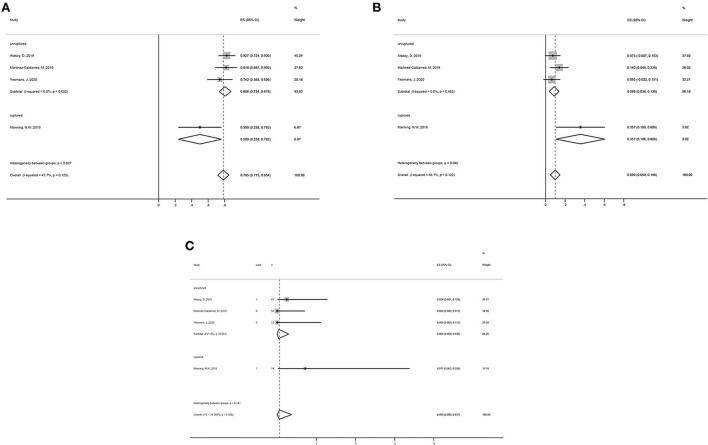
Subgroup analysis forest plots: Subgroup analysis on the **(A)** adequate occlusion rate, **(B)** morbidity rate, and **(C)** mortality rate, all categorized by the status of aneurysms (unruptured vs. ruptured group).

Subgroup analysis showed that, in the unruptured aneurysm group, the adequate occlusion rate was 80.6% (ES = 80.6%, 95% CI: 73.4–87.8%, I^2^ = 0%, *p* = 0.652; Figure 3A), the morbidity rate was 8.8% (ES = 80.6%, 95% CI: 3.8–13.8%, I^2^ = 0%, *p* = 0.463; Figure 3B), and the mortality rate was 0.4% (ES = 0.4%, 95% CI: 0.0–3.0%, I^2^ = 0%, *p* = 0.001; Figure 3C). The adequate occlusion rate, morbidity rate, and mortality rate in the ruptured aneurysm group were 50.0, 35.7, and 7.1%, respectively (Figure 3). Although the overall tendencies are noteworthy, the evidence is insufficient to draw any final conclusions.

## Discussion

FDs are new important tools for treating intracranial aneurysms (18). Considering the novelty of these devices, the risk of thromboembolic events post-implant remains a concern. It is known that patients who have undergone flow shunt placement should be treated with prolonged dual antiplatelet therapy (DAPT) to prevent thrombosis. The pipeline shield is a surface-coated device that improves the hemocompatibility of PEDs and has been shown to reduce surface platelet and fibrin adhesion as well as thrombin generation (4, 7, 19). In our review, these benefits were indirectly verified. Compared to PEDs without shield technology (11), the pipeline shield was found to be associated with higher adequate occlusion and lower mortality rates (Table 3).

**Table 3:** Comparison between PED with shield technology and the PED without shield technology.

	**PED with shield technology**	**PED without shield technology (11)**
Occlusion rate	80.9%	76.8%
Complication rate	11.1%	1.4%
Mortality rate	0.7%	0.7%

PED, pipeline embolization device.

Few studies were controlled according to the rupture status of the aneurysms. In fact, the primary treatment for ruptured aneurysms, including antiplatelet and endovascular therapies, differs from that for unruptured aneurysms. For unruptured aneurysms, in addition to encouraging patients to quit smoking and control their blood pressure, clinical decisions are made using PHASES and unruptured intracranial aneurysm treatment scores (20). Unruptured aneurysms show that short-term growth should be treated rapidly (21). Ruptured aneurysms must be treated surgically. In these patients, in addition to basic supportive care, early aneurysm occlusion is critical (22, 23). The choice of treatment depends on the overall condition of the patient, the characteristics of the aneurysm, the presence of associated hematomas and mass effects, and the overall microsurgical and endovascular expertise of the treatment center.

The pipeline shield appears to have similar outcomes to those of other well-established and more widely used FDs. In a study evaluating Silk FDs, Florez et al. reported a mortality rate of 2.8%, total thromboembolic complication rate of 6.06%, and complete aneurysm occlusion rate of 80.4% (24). In another systematic review, the rate of complete or near-total occlusion of small intracranial aneurysms treated with a Silk Vista Baby FD was 72.1% at early follow-up. The postoperative mortality rate was 2.5%, including neurological death in three cases (1.8%) (25). Asnafi et al. reported that the rate of midterm complete occlusion of the Woven EndoBridge device was 22% in an unruptured aneurysm group compared with 45% in a ruptured group. Perioperative morbidity was 4%, and perioperative mortality was 1% (26). In a meta-regression

analysis predicting aneurysm treatment outcomes with PEDs, the estimated aneurysm occlusion rate was 76%, and the estimated death and modified Rankin Scale ≤ 2 rates at unspecified follow-up times were 2 and 92%, respectively (27). Wakhloo et al. performed a study evaluating Surpass devices and found intraprocedural in-stent clot formation in 3.7% of patients. The overall morbidity rate was 6%, and the mortality rate was 2.7% (28). In another systematic review on the utilization of pipeline flex devices for treating unruptured intracranial aneurysms, a low periprocedural risk of death (0.8%) or major complications (1.8%) was reported. The risk of major complications occurring was significantly higher for large/giant aneurysms (4.4%) than for small aneurysms <10 mm (0.9%) (29). Bhatia et al. performed a systematic review on the utilization of flow redirection endoluminal devices for treating intracranial aneurysms and reported that the occlusion rate between 4 and 6 months was 73.8%, the overall reported morbidity rate was 3.9%, and procedure-related mortality was 1.4%. Complication rates fell into five categories: technical (3.6%), ischemic (3.8%), thrombotic or stenotic (6%), hemorrhagic (1.5%), and non-neurological (0.8%) (30). The DED is another surface-modified FD. In a meta-analysis of its utilization, the rate of periprocedural ischemic and hemorrhagic complications was 4.9%, the complete angiographic occlusion rate was 81.4%, and the mortality rate was 2.1% (31). Moreover, Li et al. performed a meta-analysis on the outcome of FDs with surface modifications and determined that the rate of aneurysm occlusion was 80.5% at 6 months and 85.6% at 12 months. The pooled estimate for the total ischemia rate was 6.7%, of which the severe ischemia rate was 1.8%. Morbidity and mortality rates were 6.0 and 0.7%, respectively (32).

When we collated the data, we found that some aneurysms were treated using adjunctive devices in addition to FDs, but details about the patients requiring adjunctive devices were not provided; thus, we could not analyze whether such devices were beneficial. However, in a study on pipeline-assisted coiling vs. pipeline in FDs for treating intracranial aneurysms, the authors reported that joint PED and coiling were safe with no increase in complications when compared with PED alone. Aneurysm occlusion rates and functional outcomes with PED and coiling remained comparable to those of treatment with PED alone (33). Atassoy et al. purported that putative occlusion rate differences were unlikely to be caused by a difference in adjunctive coiling (10). The rates of adjunctive coil use did not appear beneficial for aneurysm occlusion, and evidence for potential benefits is currently lacking (33). Interestingly, adjunctive coiling may be more helpful for preventing aneurysm rupture during thrombosis than for increasing the occlusion rate. Moreover, additional overlapping devices may increase coverage by increasing mesh density, thereby affecting occlusion rate. In endovascular treatments, the aneurysm sac diameter may influence the occlusion rate, especially in aneurysm coiling. As mentioned above, however, a meta-analysis on FDs revealed no relationship between the sac diameter of aneurysms and occlusion rates (34). Compared with the coils alone, combining other techniques can treat complex aneurysms and reduce the recurrence rates. In a study by Lin et al., coils in conjunction with a PED yielded higher aneurysm occlusion rates and reduced the need for retreatment (35). Because FDs cannot provide direct dome protection, large and giant aneurysms could take longer to completely occlude when treated with percutaneous endovascular embolization alone (36). Therefore, until total occlusion is achieved, these aneurysms remain at risk of rupture during the follow-up period (37, 38). In addition, studies have found intraoperative device prolapse and postoperative device displacement/shortening (39, 40), which may lead to rupture and the need for retreatment (40). Therefore, for aneurysms at risk of imminent rupture, the combined use of coils and PEDs may be more effective and provide additional mechanical support, thereby reducing the risk of device dislocation and need for retreatment.

In a meta-analysis evaluating the efficacy of FDs in posterior compared to anterior circulation aneurysms, posterior circulation aneurysms were found to be effectively treated using FDs, with comparable occlusion rates to those in anterior circulation aneurysms. However, the risk of periprocedural complications was not negligible (41). Early studies have reported higher complication rates associated with the use of FDs in the posterior circulation (42–45). This may be due to the presence of numerous perforating arteries supplying the brainstem (46). We could not compare the treatment effects between anterior and posterior circulation aneurysms because we were unable to obtain more detailed information.

Owing to the complexity of patients' conditions and disagreements on antiplatelet regimens for pipeline shield utilization, protocols for antiplatelet therapy among the trials included in our review were not uniform (Table 4). The FDs need DAPT to prevent thrombosis and ischemic complications. However, DAPT increases the risk of hemorrhagic complications (47). Studies have shown that the pipeline shield can reduce platelet adhesion to the surface (19, 48, 49). *In vivo*, single antiplatelet therapy with pipeline shield had similar thrombogenicity to that of DAPT with PED-Flex (4). Therefore, pipeline shield devices may reduce the need for antiplatelet drugs, thereby reducing the risk of hemorrhage. The role of antiplatelet and anticoagulant medications in treating unruptured aneurysms has been controversial. Retrospective studies have reported that patients taking long-term aspirin exhibit a reduced risk of rupture, while those taking dipyridamole and new aspirin may be at risk of subarachnoid hemorrhage (50, 51). In another study, patients taking aspirin (28%) were found to have lower bleeding rates than those not taking aspirin (40%) (52). Aspirin was also not found to worsen outcomes after subarachnoid hemorrhage (51). In contrast, anticoagulants were associated with poor prognosis after subarachnoid hemorrhage (53) but did not increase the risk of aneurysm rupture (54, 55).

**Table 4:** Antiplatelet regimen in each study.

**Study, year**	**Antiplatelet**	**Platelet-resistance testing**
Atasoy et al. (10)	DAPT 7–10 days preprocedure, continued clopidogrel once daily for 6–9months and continued aspirin for life (all doses, 75mg daily).	No.
Manning et al. (17)	14/14(100%) patients received SAPT therapy. 2/14(14%) patients were preloaded, and 2/14(14%) patients were loaded immediately postoperatively. The remaining 10/14(71%) patients were loaded intraoperatively.	Not mentioned.
Martinez-Galdamez et al. (12)	Prior to the procedure, 46/50 (92%) patients received DAPT (aspirin +clopidogrel/prasugrel) and 4/50 (8%) patients received SAPT (clopidogrel). 50/50 (100%) patients were prescribed DAPT between ≥1 month and ≤ 1 year post-procedure.	Not mentioned.
Pikis et al. (13)	31/33(94%) patients received DAPT (aspirin 100 mg/day +clopidogrel 75 mg/day) 5 days preprocedure. 1/33(3%) patient received DAPT (aspirin 100 mg/day +prasugrel 10 mg/ day) 5 days preprocedure. 1/33(3%) patient received SAPT (prasugrel 10 mg/ day) 5 days preprocedure. All patients were instructed to continue with the preprocedural antiplatelet regimen until the 6 month angiographic and clinical follow-up.	No.
Rice et al. (14)	195/205 (95.6%) patients received antiplatelet therapy prior to study treatment. DAPT was administered pre- procedure (≥7 days) in 57/195 (29.2%) of subjects, on days 1–6 preprocedure in 104/195 (53.3%), on the day of the procedure in 182/195 (93.3%), and immediately prior to the procedure in 161/195 (82.6%). 193/195 (99%) subjects received DAPT post- procedure, and of these, 20% (39/195) interrupted DAPT within 3 months and continued with SAPT [either aspirin (19.5%) or clopidogrel (0.5%)]. 24/195(12.3%) subjects never interrupted DAPT during follow-up. SAPT was administered pre- procedure (≥7 days) in 4/195 (2.1%) of subjects, on days 1–6 pre- procedure in 9/195 (4.6%), on the day of the procedure in 8/195 (4.1%), and immediately pre- procedure in 13/195 (6.7%). Only 2/195 (1.0%) of subjects received SAPT post- procedure.	Not mentioned.
Trivelato et al. (15)	Patients were asked to take DAPT (aspirin 100 mg/day+ clopidogrel 75 mg/day or ticagrelor 90 mg twice a day) for 5 days prior to the intervention and for 6 months afterward. Aspirin was maintained for another 6 moonths. For ruptured aneurysms, all patients were premedicated with a loading dose of aspirin (300 mg) plus clopidogrel (600 mg) 3 h before the procedure. After treatment, these patients received the standard antiplatelet regimen.	No.
Yeomans et al. (16)	The elective cases received dual antiplatelet therapy post-procedure. The acute cases received single antiplatelet therapy post-procedure. Elective patients received single oral doses of aspirin 300 mg and clopidogrel 600 mg the night before the procedure. The VerifyNow P2Y12 assay (Werfen, Spain) was used to confirm an adequate response to dual antiplatelet therapy. All unruptured, elective aneurysm patients with a good P2Y12 antagonist response were placed on a post-procedure regimen of oral clopidogrel 75 mg once daily for 5 months and oral aspirin 75 mg once daily for 12 months. The procedure would have been abandoned in P2Y12 antagonist non-responders. Poor P2Y12 antagonist responders would have been given oral prasugrel 5–10 mg once daily for 5 months. Acute patients received a single intravenous dose of aspirin 500 mg immediately prior to the deployment of the Pipeline device during the procedure. All acute patients received a single antiplatelet therapy regimen post-procedure of oral aspirin 75 mg once daily for 12 months.	VerifyNow P2Y12 assay.

DAPT, dual antiplatelet therapy; SAPT, single antiplatelet therapy; IV, intravenous injection.

Our study has the following limitations. As some articles included in our review reported retrospective results based on small samples, our results may be biased. Further, as antiplatelet therapy regimens vary between studies and institutions, no reliable conclusions could be drawn regarding antiplatelet therapy.

## Conclusion

Technological improvements have greatly improved endovascular treatment options for aneurysms. As a novel surface-modified PED, the pipeline shield is increasingly used to treat intracranial aneurysms. From our review, we determined that this intervention results in low rates of mortality and a high rate of occlusion.

## Data availability statement

The original contributions presented in the study are included in the article/supplementary material, further inquiries can be directed to the corresponding authors.

## Author contributions

CL and LJin conceived the project and drafted the manuscript. EL, BL, and ZF searched the databases and analyzed data. JD, SY, PL, and LJia were responsible for the whole process of supervision. SZ and WH revised the manuscript. All authors read and approved the final version of the manuscript.

## Funding

This work was supported by grants from the National Natural Science Foundation of China (Grant Number 81560227) and the Yunnan Health Training Project of High-level Talents (Grant Number H-2017030).

## Conflict of interest

The authors declare that the research was conducted in the absence of any commercial or financial relationships that could be construed as a potential conflict of interest.

## Publisher's note

All claims expressed in this article are solely those of the authors and do not necessarily represent those of their affiliated organizations, or those of the publisher, the editors and the reviewers. Any product that may be evaluated in this article, or claim that may be made by its manufacturer, is not guaranteed or endorsed by the publisher.
